# Regional Differences in Ca^2+^ Signaling and Transverse-Tubules across Left Atrium from Adult Sheep

**DOI:** 10.3390/ijms24032347

**Published:** 2023-01-25

**Authors:** Caroline Cros, Matthieu Douard, Sebastien Chaigne, Come Pasqualin, Gilles Bru-Mercier, Alice Recalde, Caroline Pascarel-Auclerc, Thomas Hof, Michel Haïssaguerre, Meleze Hocini, Pierre Jaïs, Olivier Bernus, Fabien Brette

**Affiliations:** 1 INSERM U1045, Centre de Recherche Cardio-Thoracique de Bordeaux, Université de Bordeaux, 33000 Bordeaux, France; 2 IHU Liryc, Electrophysiology and Heart Modeling Institute, Fondation Bordeaux Université, 33000 Bordeaux, France; 3 Cardiac Electrophysiology and Cardiac Stimulation Team, Bordeaux University Hospital (CHU), 33000 Bordeaux, France; 4 EA4245, Transplantation, Immunologie et Inflammation (T2I), Groupe PCCV, Université de Tours, 37000 Tours, France

**Keywords:** cardiac myocyte, atria, calcium, t-tubule, excitation-contraction coupling, large animal model

## Abstract

Cardiac excitation-contraction coupling can be different between regions of the heart. Little is known at the atria level, specifically in different regions of the left atrium. This is important given the role of cardiac myocytes from the pulmonary vein sleeves, which are responsible for ectopic activity during atrial fibrillation. In this study, we present a new method to isolate atrial cardiac myocytes from four different regions of the left atrium of a large animal model, sheep, highly relevant to humans. Using collagenase/protease we obtained calcium-tolerant atrial cardiac myocytes from the epicardium, endocardium, free wall and pulmonary vein regions. Calcium transients were slower (time to peak and time to decay) in free wall and pulmonary vein myocytes compared to the epicardium and endocardium. This is associated with lower t-tubule density. Overall, these results suggest regional differences in calcium transient and t-tubule density across left atria, which may play a major role in the genesis of atrial fibrillation.

## 1. Introduction

Excitation-contraction (EC) coupling is the physiological process that links electrical excitation with contraction in cardiac myocytes [1]. Excitation-contraction coupling begins when an action potential depolarizes the sarcolemma of the cardiac myocyte and opens voltage-gated ion channels, allowing calcium (Ca^2+^) entry into the cell through L-type Ca^2+^ channels. This extracellular Ca^2+^ influx triggers the release of additional Ca^2+^ from the internal Ca^2+^ stores of the sarcoplasmic reticulum (SR) via a process termed ‘Ca^2+^-induced Ca^2+^-release’ (CICR) [2]. The consequent systolic Ca^2+^ transient, which activates the contractile machinery within the heart muscle cells, is the spatial and temporal sum of such local Ca^2+^ releases. Relaxation occurs by the reuptake of Ca into the SR via the SR Ca^2+^ ATPase (SERCA) and extrusion from the cell via the sodium calcium exchanger (NCX).

In ventricular myocytes, invaginations of the sarcolemma, transverse (t-) tubules, play a key role in EC coupling [3]. L-type Ca^2+^ channels are concentrated in t-tubules (vs. surface membrane) and are closely juxtaposed to clusters of Ryanodine receptors. This ensures efficient and coordinated Ca^2+^ release throughout the cell.

In contrast, in atrial myocytes t-tubules were thought to be lacking or poorly developed. However, in the last ten years several studies have demonstrated the existence of a t-tubular network in large animal species (dog, cow, horse, sheep, pig, human) [4]. More recently, super-resolution confocal imaging revealed the presence of a t-tubule network in atrial myocytes from rodents (rat and mouse) [5].

The disruption in Ca^2+^ homeostasis has been implicated in arrhythmia via afterdepolarization as well as reentry [6]. Such events could be due to a difference in t-tubular organization. The disruption of the t-tubule network in ventricular myocytes is well demonstrated in heart failure [7]. In atrial fibrillation, which is the most common type of heart arrhythmia, investigations have begun to examine the role of Ca^2+^ homeostasis and the t-tubule network [8,9]. Studies are hampered by the requirement for animal models that approach the human condition. Sheep are considered a reliable animal model for chronic atrial fibrillation [8,10]; however, similar to dogs [11] and pigs [12], most studies investigate myocytes from right atria and/or left atria as a whole. Given the crucial role of the pulmonary vein cardiac muscle sleeve in the initiation and conduction of ectopic electrical activities [13], it is essential to investigate regional EC coupling in left atria from an animal model relevant to humans, including pulmonary vein cardiac myocytes. To date, limited studies in rodents have given information on Ca^2+^ cycling in cardiac cells from the pulmonary vein [14,15]. However, rodent models are unable to recapitulate an atrial fibrillation phenotype that resembles human atrial fibrillation, and large animal models are the gold standard to better represent human atrial fibrillation to advance understanding that may be translated to clinical care [16].

We hypothesize that Ca^2+^ signaling is different in specific regions of the left atrium. To test this hypothesis, we developed a method to isolate atrial cardiac myocytes from four different regions of the left atrium from sheep and investigated Ca^2+^ homeostasis and the t-tubular network.

## 2. Results and Discussion

### 2.1. Regional Left Atria Myocyte Isolation

To our knowledge, the left atrium has been mainly studied as an entity and no regionalized cell isolation has been attempted. In preliminary experiments, we used a cardioplegic solution containing (in mM): 110 NaCl, 16 KCl, 16 MgCl_2_, 10 NaHCO_3_, 10 glucose and 1.2 CaCl_2_, supplemented with heparine (0.5 mL/L) to rinse the heart after excision but the yield and Ca^2+^ tolerance of the cardiac myocytes were very poor. By changing to cold Ca^2+^-free cell isolation solution (see Section 3.2), we were able to obtain good yield (>50%) and Ca^2+^-tolerant cardiac myocytes from four regions of the left atrium of the sheep heart. More than 80% of the cardiac myocytes in each region were responsive to electrical field stimulation (contraction) indicating good cell health. Therefore, we next assessed whether Ca^2+^ homeostasis and the t-tubule network were homogeneous across its different regions in the left atrium.

### 2.2. Ca^2+^ Signaling Heterogeneity over the Left Atrium

**Figure 1:**
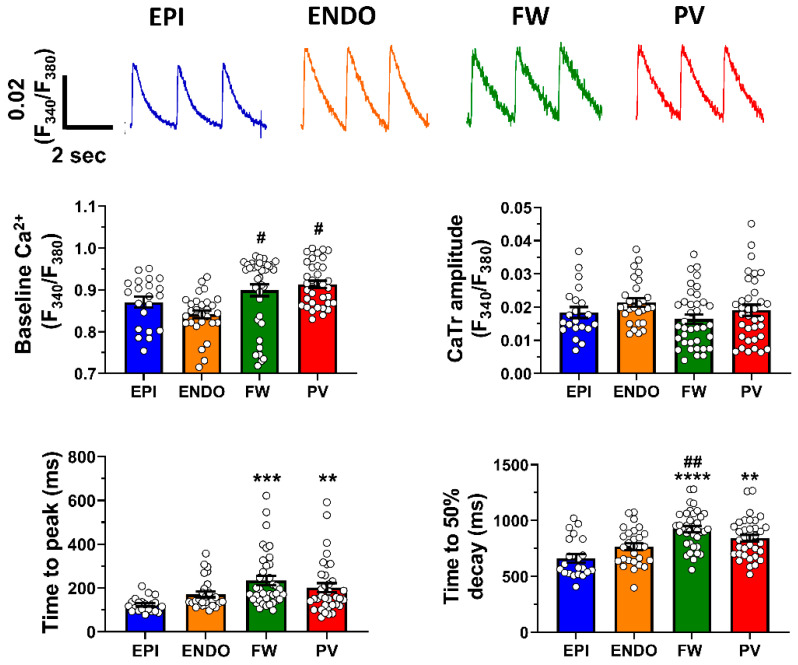
Ca^2+^ transient heterogeneity in left atria from sheep. Top panel shows representative Ca^2+^ transient (CaTr) recorded at 0.5 Hz in adult sheep cardiac myocytes isolated from the epicardium (EPI) and endocardium (ENDO) regions of the left appendage (blue and orange traces, respectively); the free wall region (FW) and the pulmonary vein (PV) region of the left atrium (green and red traces, respectively). Lower panel is Mean ± SEM diastolic Ca^2+^, CaTr amplitude, time to peak and time to 50% transient decay recorded in those four atrial regions. n = 21, 28, 37 and 35 cells for EPI, EnNDO, FW and PV groups, respectively. Data are represented as mean ± SEM with ** *p* < 0.01, *** *p* < 0.001, **** *p* < 0.0001 compared to EPI and # *p* < 0.05, ## *p* < 0.01 compared to ENDO.

We first used ratiometric dye (Fura-2) to estimate [Ca^2+^]_i_, as it is not affected by probe loading, bleaching and/or leakage. This allows the comparison of values between different regions and importantly true resting Ca^2+^ value (diastolic). Figure 1 (top panel) shows representative Ca^2+^ transients recorded from myocytes isolated from the left atrial epicardium and endocardium appendage region (EPI, blue trace and ENDO, orange trace, respectively), the free wall region (FW, green trace) and the pulmonary vein region (PV, red trace).

The mean data presented in Figure 1 (lower panel) indicate that diastolic Ca^2+^ is significantly higher in the FW and PV compared to the appendage (EPI and ENDO). Interestingly, the amplitude of the Ca^2+^ transient was similar in all regions. In contrast, the FW and myocytes from pulmonary show slower kinetics of the Ca^2+^ transient for the time to peak and time to 50% decay. The slower time to peak suggests a less efficient EC coupling in FW and PV cardiac myocytes compared to EPI and ENDO. The slower time to 50% decay could be due, at least in part, to the less efficient extrusion of Ca^2+^ from the cytosol via the NCX. Overall, these data demonstrated that the left atrium displays the region-specific kinetics of Ca^2+^ signaling and differences in diastolic Ca^2+^ but similar Ca^2+^ transient amplitude. Together, these results suggest a difference in t-tubule density because t-tubules are crucial for efficient EC coupling [17] and NCX is concentrated within t-tubules [18].

### 2.3. Heterogeneity of T-Tubule Network across the Left Atrium

**Figure 2:**
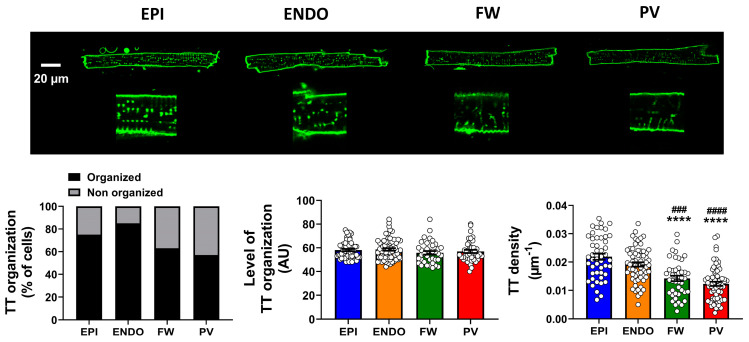
T-tubule characteristics in left atria from sheep. Top panel shows representative images of atrial myocytes stained with di-8 ANNEPS and visualized under confocal microscopy from four regions, epicardium (EPI), endocardium (ENDO), free wall (FW) and pulmonary vein (PV). Images of ×2.8 magnification are shown (below). Lower panel is percentage of cells showing an organized t-tubule (TT) network (black bars) and no organized TT (grey bars), Mean ± SEM TT regularity in cells with organized TT network and associated TT density in each group of cells. Data for TT organization and regularity analysis are from EPI = 81; ENDO = 79; FW = 59 and PV = 76. Data for TT density analysis are from EPI = 48; ENDO = 63; FW = 43 and PV = 65. **** *p* < 0.0001 compare to EPI and ### *p* < 0.001, #### *p* < 0.0001 compare to ENDO.

Staining the cell membrane with di-8-ANEPPS, confocal images revealed the presence of tubular structures in the four regions of the left atrium (Figure 2).

However, the percentage of cells displaying an organized t-tubular network varied across atrial regions with EPI and ENDO showing a larger proportion of cells with organized t-tubules and the PV presenting the lower (57%) proportion of cells with organized t-tubules (Figure 2, lower panel). Among the cells that displayed a tubular structure, the level of t-tubule organization was relatively high in all regions and no significant difference was observed for the index of regularity (Figure 2, lower panel). In contrast, further quantification revealed that the density of the t-tubules was significantly lower in the PV and FW compared to the EPI and ENDO regions (Figure 2, lower panel). The molecular mechanisms involved in t-tubule formation and maintenance, thus density, remain unclear [7].

Overall, these data clearly show that the cardiac myocytes from the PV and FW regions present a less organized t-tubule network, which could explain the difference in Ca^2+^ transient kinetics observed in Figure 1.

To investigate this further, we loaded cells with Fluo-4 AM and used 2D confocal microscopy to record images of Ca^2+^ transients from the whole cell at 30 fps. Such experiments have identified regional Ca^2+^ dynamics in Purkinje fibers, which lack t-tubule networks [19]. Videos in Supplementary Materials show that electrical stimulation induced an increase in Ca^2+^ across the whole width of the cell in all four regions (EPI, ENDO, FW, PV; Videos S1–S4). Under our experimental conditions, we were not able to detect the propagation of Ca^2+^ from the surface membrane to the cell interior, as is the case in detubulated or cardiac myocytes with a lower density of t-tubules [3]. These results indicate that Ca^2+^ spatially rises homogenously at a 33 ms interval (our sampling frequency, 30 fps) despite differences in t-tubule density between regions of the left atrium. This is the first limitation of our study. More subtle differences in spatial Ca^2+^ inhomogeneities may be uncovered using a higher speed confocal microscope. Another limitation of our study is the fact that we have not specifically observed cellular arrhythmic events in the four regions studied. However, based on the literature on Ca^2+^ signaling and cardiac arrythmia [20], we can speculate that the observed differences in Ca^2+^ handling may be related to the potency of the PV myocytes to trigger AF. Indeed, in multicellular preparations, an association between Ca^2+^ cycling and arrhythmia has been shown in dogs [21] and mice [22]. Finally, this study focused specifically on the left atrium, which is the primary site for AF triggering (introduction). The right atrium, especially the region of the superior vena cava, has also been identified as one of the most common sources of non-pulmonary vein triggering for AF. Future research may provide a complete mapping of Ca^2+^ and t-tubule density in the right atrium, as recently achieved in mice [23].

## 3. Materials and Methods

### 3.1. Animal Ethics

This study was carried out in accordance with the EU Directive 2010/63/EU for protection of animals used for scientific purposes and approved by the local ethical authorities (CEEA50) at University of Bordeaux, France.

### 3.2. Left Atria Cardiomyocyte Isolation

**Figure 3:**
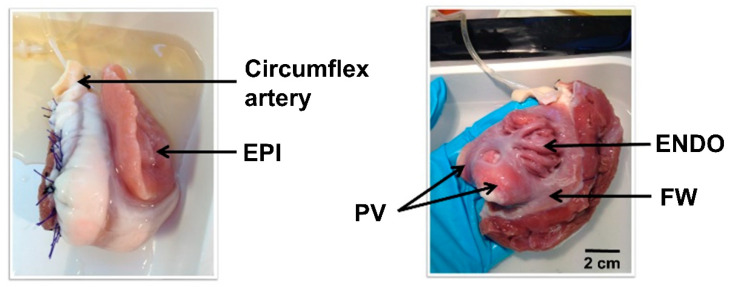
Langendorff left atrial preparation. Perfusion through the circumflex artery after suture of the leaky branches was performed to be able to dissociate atrial cardiac myocytes from epicardium (EPI), endocardium (ENDO), Free Wall (FW) and pulmonary vein (PV) regions.

Hearts were obtained from young female adult sheep (18–24 months, N = 24, 40–50 kg). Sheep were pre-medicated with intramuscular injection of ketamine (20 mg/kg) and acepromazine (0.02 mL/kg). Anesthesia was induced with intravenous injection of sodium pentobarbital (10 mg/kg). Then, sheep were intubated and ventilated with 100% oxygen and 2–3% isoflurane to maintain anesthesia. Heparin (2 mg/kg) was injected intravenously to prevent blood coagulation. Sheep were euthanized by intravenous injection of pentobarbital (30 mL/50 kg) and the healthy heart was rapidly excised. The aorta was cannulated and the heart was rinsed with cold Ca^2+^-free cell isolation solution (4 °C) (in mM): 130 NaCl, 5.4 KCl, 1.4 MgCl_2_, 0.4 NaH_2_PO_4_, 5 HEPES, 10 glucose, 10 creatine, 20 taurine (pH 7.6 with NaOH) supplemented with heparine (0.5 mL/L). The right atrial tissue was cut and the ventricles were removed. The left coronary artery (circumflex) was then cannulated and mounted into a Langendorff perfusion system after suture of the leaky atrial branches. Atrial tissue was perfused with isolation solution (20 min at 20 mL/min) described above without heparine. Perfusion was then continued with Ca^2+^-free isolation solution supplemented with 0.1 mM EGTA for ~20 min, followed by perfusion isolation solution containing 1 mg/mL collagenase (type II, Worthington, Lakewood, NA, USA), 0.1 mg/mL protease (type XIV, Sigma, St. Louis, MO, USA) and 0.08 mM Ca^2+^ and recirculated for ~30 min. The enzymes were washed out with isolation solution containing 0.2 mM Ca^2+^ for ~5 min. Left atrium (LA) was removed and the left appendage epicardium (EPI), endocardium (ENDO), LA free wall (FW) and pulmonary vein (PV) regions were separated (Figure 3), cut into small pieces and further dissociated into single cells by gently agitating the muscle pieces.

Cells were stored in a low Ca^2+^ solution (0.75 mM) at room temperature and used within 8 h after isolation. Cardiomyocyte isolation was performed at 37 ± 1 °C.

### 3.3. Whole Cell Ca^2+^ Transient Recording

Cardiomyocytes were loaded with the Ca^2+^-sensitive fluorescent indicator Fura 2-AM (4 µM, Invitrogen, Carlsbad, CA, USA) for 10 min at room temperature and then rinsed with control Tyrode solution containing (in mmol/L): 137 NaCl, 5 KCl, 1.8 CaCl_2_, 1 MgCl_2_, 10 glucose and 20 HEPES (pH 7.4 with NaOH). Cells were placed in a perfusion chamber mounted on the stage of an inverted microscope (Nikon Eclipse Ti, Invitrogen, Carlsbad, CA, USA) and perfused with Tyrode solution with experimental bath temperature maintained at 37 ± 1 °C via a feedback-controlled heater system (Cell Micro Controls, Norfolk, VA, USA). Cells were electrically field stimulated at 0.5 Hz with a pair of platinum electrodes. Fura-2 fluorescence was elicited by alternate illumination with 340 and 380 nm light obtained using a monochromator (Optoscan Fluorescence System, Cairn Research, Faversham, UK) in front of a Xenon excitation lamp. The fluorescence emitted at 510 nm was monitored using a photomultiplier tube (Cairn Research, Faversham, UK). The ratio (F_340_/F_380_) was used as an index of [Ca^2+^]_i_. IonWizard software (IonOptix, Westwood, MA, USA) was used for recordings.

### 3.4. Cell Membrane Staining

Cell membrane was visualized by staining with the lipophilic dye di-8-ANNEPS (5 µM for 2 min). The cells were then resuspended in Tyrode solution (above) and imaged using confocal laser scanning microscopy (Olympus France, Rungis, France) using 488 nm excitation light with detection at >505 nm. Representative confocal images are shown in the x-y plane midway through the cell.

### 3.5. Rapid 2D Ca^2+^ Imaging

Single cells were loaded with the fluorescent Ca^2+^ dye fluo-4 AM (10 µM, 30 min) at room temperature. A further 30 min was allowed for washout of fluo-4 AM and de-esterification. Cells were placed on the stage of a motorized inverted microscope (Olympus IX83, Olympus France, Rungis, France, objective 30× silicone immersion) and field stimulated at 0.5 Hz. Fluorescence of Fluo-4 was imaged by 2D spinning disk confocal microscopy unit Yokogawa CSU-W1 (Yokogawa, Tokyo, Japan) coupled with EMCCD camera (iXon ultra 897, Andor, Belfast, UK) at 30 frames per second.

### 3.6. Data Analysis

Whole cell Ca^2+^ transient data were analyzed using IonWizard (IonWizard software (Version 6.3, Ionoptix, Westwood, MA, USA) by averaging 3 signals at steady state. Transverse tubules (TT) were quantified in a blinded fashion using ImageJ software (version 1.5, Bethesda, MD, USA)and plugin TTorg [24]. This plugin allows the quantification of t-tubule organization (% of cells) and level of t-tubule organization (in arbitrary unit, AU). T-tubule density (in µm^−1^) was assessed with the calculation of the ratio: t-tubule length/cell surface. Two-dimensional calcium imaging movies were reconstructed from MetaMorph MetaMorph (version 7.8, Molecular Devices, San Jose, CA, USA) images using ImageJ.

### 3.7. Statistics

Data are presented as mean ± SEM. GraphPad Prism (GraphPad Software (version 7.04, (GraphPad Software, Boston, MA, USA)) was employed for the statistical analysis and graph creation. Datasets were tested for normality using Shapiro–Wilk test. One-way analysis of variance (ANOVA) with Dunnet’s multiple test was used to compare normally distributed datasets and Kurskal–Wallis with Dunn’s multiple test was used to compare non-normal data distributions. *p* < 0.05 was taken as significant.

## 4. Conclusions

We developed a method to isolate cardiac myocytes from four different regions of the left atrium in an animal model relevant to the study of human atrial fibrillation. Importantly, we were able to characterize Ca^2+^ homeostasis and t-tubule density in cardiac myocytes from the pulmonary vein region, which is crucial in triggering ectopic activity. The lower t-tubule density is prone to arrhythmogenesis, even in control conditions [25]. Our approach should be useful for a better understanding of chronic atrial fibrillation.

## Data Availability

The data supporting the study findings can upon reasonable request be made available from the corresponding author.

## Supplementary Materials

The following supporting information can be downloaded at: https://www.mdpi.com/article/10.3390/ijms24032347/s1.

## References

[1] Bers D.M. (2002). Cardiac excitation-contraction coupling. Nature.

[2] Fabiato A. (1983). Calcium-induced release of calcium from the cardiac sarcoplasmic reticulum. Am. J. Physiol..

[3] Brette F., Orchard C. (2003). T-tubule function in mammalian cardiac myocytes. Circ. Res..

[4] Dibb K.M., Clarke J.D., Horn M.A., Richards M.A., Graham H.K., Eisner D.A., Trafford A.W. (2009). Characterization of an extensive transverse tubular network in sheep atrial myocytes and its depletion in heart failure. Circ. Heart Fail..

[5] Brandenburg S., Kohl T., Williams G.S., Gusev K., Wagner E., Rog-Zielinska E.A., Hebisch E., Dura M., Didie M., Gotthardt M. (2016). Axial tubule junctions control rapid calcium signaling in atria. J. Clin. Investig..

[6] Schotten U., Verheule S., Kirchhof P., Goette A. (2011). Pathophysiological mechanisms of atrial fibrillation: A translational appraisal. Physiol. Rev..

[7] Setterberg I.E., Le C., Frisk M., Li J., Louch W.E. (2021). The Physiology and Pathophysiology of T-Tubules in the Heart. Front. Physiol..

[8] Lenaerts I., Bito V., Heinzel F.R., Driesen R.B., Holemans P., D’Hooge J., Heidbuchel H., Sipido K.R., Willems R. (2009). Ultrastructural and functional remodeling of the coupling between Ca2+ influx and sarcoplasmic reticulum Ca^2+^ release in right atrial myocytes from experimental persistent atrial fibrillation. Circ. Res..

[9] Macquaide N., Tuan H.T., Hotta J., Sempels W., Lenaerts I., Holemans P., Hofkens J., Jafri M.S., Willems R., Sipido K.R. (2015). Ryanodine receptor cluster fragmentation and redistribution in persistent atrial fibrillation enhance calcium release. Cardiovasc. Res..

[10] Martins R.P., Kaur K., Hwang E., Ramirez R.J., Willis B.C., Filgueiras-Rama D., Ennis S.R., Takemoto Y., Ponce-Balbuena D., Zarzoso M. (2014). Dominant frequency increase rate predicts transition from paroxysmal to long-term persistent atrial fibrillation. Circulation.

[11] Arora R., Aistrup G.L., Supple S., Frank C., Singh J., Tai S., Zhao A., Chicos L., Marszalec W., Guo A. (2017). Regional distribution of T-tubule density in left and right atria in dogs. Heart Rhythm.

[12] Gadeberg H.C., Bond R.C., Kong C.H.T., Chanoit G.P., Ascione R., Cannell M.B., James A.F. (2016). Heterogeneity of T-Tubules in Pig Hearts. PLoS ONE.

[13] Haissaguerre M., Jais P., Shah D.C., Takahashi A., Hocini M., Quiniou G., Garrigue S., Le Mouroux A., Le Metayer P., Clementy J. (1998). Spontaneous initiation of atrial fibrillation by ectopic beats originating in the pulmonary veins. N. Engl. J. Med..

[14] Okamoto Y., Takano M., Ohba T., Ono K. (2012). Arrhythmogenic coupling between the Na+–Ca2+ exchanger and inositol 1,4,5-triphosphate receptor in rat pulmonary vein cardiomyocytes. J. Mol. Cell. Cardiol..

[15] Pasqualin C., Yu A., Malécot C.O., Gannier F., Cognard C., Godin-Ribuot D., Morand J., Bredeloux P., Maupoil V. (2018). Structural heterogeneity of the rat pulmonary vein myocardium: Consequences on intracellular calcium dynamics and arrhythmogenic potential. Sci. Rep..

[16] Schüttler D., Bapat A., Kääb S., Lee K., Tomsits P., Clauss S., Hucker W.J. (2020). Animal Models of Atrial Fibrillation. Circ. Res..

[17] Gomez A.M., Valdivia H.H., Cheng H., Lederer M.R., Santana L.F., Cannell M.B., McCune S.A., Altschuld R.A., Lederer W.J. (1997). Defective excitation-contraction coupling in experimental cardiac hypertrophy and heart failure. Science.

[18] Despa S., Brette F., Orchard C.H., Bers D.M. (2003). Na/Ca exchange and Na/K-ATPase function are equally concentrated in transverse tubules of rat ventricular myocytes. Biophys. J..

[19] Daniels R.E., Haq K.T., Miller L.S., Chia E.W., Miura M., Sorrentino V., McGuire J.J., Stuyvers B.D. (2017). Cardiac expression of ryanodine receptor subtype 3; a strategic component in the intracellular Ca(2+) release system of Purkinje fibers in large mammalian heart. J. Mol. Cell Cardiol..

[20] Landstrom A.P., Dobrev D., Wehrens X.H.T. (2017). Calcium Signaling and Cardiac Arrhythmias. Circ. Res..

[21] Patterson E., Lazzara R., Szabo B., Liu H., Tang D., Li Y.-H., Scherlag B.J., Po S.S. (2006). Sodium-Calcium Exchange Initiated by the Ca2+Transient: An Arrhythmia Trigger Within Pulmonary Veins. J. Am. Coll. Cardiol..

[22] Rietdorf K., Masoud S., McDonald F., Sanderson M.J., Bootman M.D. (2015). Pulmonary vein sleeve cell excitation–contraction-coupling becomes dysynchronized by spontaneous calcium transients. Biochem. Soc. Trans..

[23] Lang D., Medvedev R.Y., Ratajczyk L., Zheng J., Yuan X., Lim E., Han O.Y., Valdivia H.H., Glukhov A.V. (2022). Region-specific distribution of transversal-axial tubule system organization underlies heterogeneity of calcium dynamics in the right atrium. Am. J. Physiol. Heart Circ. Physiol..

[24] Pasqualin C., Gannier F., Malécot C.O., Bredeloux P., Maupoil V. (2015). Automatic quantitative analysis of t-tubule organization in cardiac myocytes using ImageJ. Am. J. Physiol. Cell Physiol..

[25] Orchard C.H., Bryant S.M., James A.F. (2013). Do t-tubules play a role in arrhythmogenesis in cardiac ventricular myocytes?. J. Physiol..

