# Supplementary material for: Regional Differences in Ca2+ Signaling and Transverse-Tubules across Left Atrium from Adult Sheep
Source: Int J Mol Sci. 2023 Jan 25;24(3):2347. doi: 10.3390/ijms24032347 (PMC9916916; doi:10.3390/ijms24032347)
Supplement: Supplementary file 1 [file ijms-24-02347-s001.zip › Ijms- 2094739-supplementary.pdf]

Animated sequence of images of the typical Ca^{2+} response to electric stimulation in 4 cells from different regions of the left atria. Frames were recorded at 30 fps. Cells were loaded with Fluo-4.

Video S1: Epicardium

Video S2: Endocardium

Video S3: Free wall

Video S4: Pulmonary vein.
